# Emodin repairs interstitial cells of Cajal damaged by cholelithiasis in the gallbladder 

**DOI:** 10.3389/fphar.2024.1424400

**Published:** 2024-09-18

**Authors:** Zhen-peng Huang, Hu Qiu

**Affiliations:** ^1^ Faculty of Nursing, Guangxi University of Chinese Medicine, Nanning, China; ^2^ Department of Oncology, Renmin Hospital of Wuhan University, Wuhan, China

**Keywords:** interstitial cells of Cajal, SCF/c-kit pathway, gallbladder, cholelithiasis, emodin

## Abstract

**Background:**

Hypercholesterolemia induces cholelithiasis and dysfunction of gallbladder motility. Interstitial cells of Cajal (ICCs) contribute to gallbladder motility. Emodin modulates the contractility of the gallbladder muscle; however, the underlying mechanism is unknown.

**Aim:**

This study aimed to explore the effects of emodin on gallbladder ICCs with cholelithiasis in a guinea pig model.

**Methods:**

Animals were randomly divided into a healthy control group and three study groups. All study groups received a high-cholesterol diet (HCD) for 8 weeks. Subsequently, they were randomly assigned to either the HCD group or one of the emodin treatment groups lasting 4 or 8 weeks. Total cholesterol (TC) and triglycerides (TG) were measured to determine changes in serum lipid levels. Immunohistochemistry was performed to detect the morphology and number of ICCs. TUNEL assays were performed to detect ICC apoptosis. Transmission electron microscopy was employed to observe ICC structure. Western blotting and real-time polymerase chain reaction were used to detect changes in stem cell factor (SCF)/c-kit pathway expression.

**Results:**

Serum TC and TG were higher in all study groups. In cases of cholelithiasis, the SCF/c-kit pathway was downregulated, the number of gallbladder ICCs decreased, apoptosis increased, and the ICC network structure was damaged. After emodin treatment, the SCF/c-kit pathway was upregulated, the number of gallbladder ICCs increased, apoptosis decreased, and the ICC network structure recovered.

**Conclusion:**

Cholelithiasis downregulates the SCF/c-kit pathway and damages gallbladder ICCs. Emodin upregulates the SCF/c-kit pathway and increases gallbladder ICCs, contributing to recovery from gallbladder motility disorders.\

## 1 Introduction

Hypercholesterolemia is a public health problem worldwide and is clinically classified as one of the main subtypes of hyperlipidemia (Li Z et al., 2022). Hypercholesterolemia is prevalent, with rates of 26.4% in Ethiopia and rates that vary between 2.5% and 48.1% in China, with a median of 8.2% (Belete AK et al., 2023; Huang Y et al., 2014; Pan J et al., 2018). Excessive cholesterol intake is not only a major risk factor for vascular disease but also induces cholelithiasis and gallbladder motility dysfunction (Di Ciaula A et al., 2019; Kakimoto T et al., 2017; Kano M et al., 2002; Schneider EH et al., 2022). The prevalence of cholesterol gallstones is common worldwide, with a 10%–15% incidence in Western countries and 4.42%–11% in China. Gastrointestinal endoscopy and surgery are common approaches to treating cholelithiasis (Cianci and Restini, 2021).

However, the mechanism underlying the dysfunction of gallbladder motility and treatment of cholelithiasis remains elusive (Stinton LM et al., 2010; Stinton LM and Shaffer EA, 2012; Wilkins T et al., 2017).

The interstitial cells of Cajal (ICCs) promote gastrointestinal smooth muscle electrical activity and regulate digestive tract neurotransmitters in the gastrointestinal tissue (Foong D et al., 2020). Gallbladder motility is regulated by the influence of gallbladder ICCs on electrical rhythmicity and contractility (Ding F et al., 2023). Recent studies, including our own research, have highlighted the significant role of gallbladder ICCs in cholelithiasis and gallbladder motility dysfunction (Ding R et al., 2019; Huang L et al., 2021; Huang ZP et al., 2018; Jung MS et al., 2022).

Emodin (1,3,8-trihydroxy-6-methylanthraquinone) is a natural anthraquinone derivative contained in many medicinal herbs, such as *Polygonum cuspidatum*, with wide use in many countries, especially in Asia (Dong X et al., 2020). Notably, it possesses anti-inflammatory, antiviral, antibacterial, antiallergic, neuroprotective, immunosuppressive, anti-osteoporotic, antidiabetic, and hepatoprotective properties (Dong X et al., 2020; Luo N et al., 2021; Mitra S et al., 2022). Emodin has been observed to influence the contractility of gastrointestinal smooth muscles, including those of the gallbladder; however, the precise mechanism remains to be fully elucidated (Wu ZX et al., 2009). Although emodin has been shown to modulate the contractility of the gallbladder muscle via ICCs, the exact mechanism remains unknown. In this study, we aimed to explore the effect of emodin on gallbladder ICCs and gallbladder contractility in an animal model.

## 2 Materials and methods

### 2.1 Animal experiments

**FIGURE 1:**
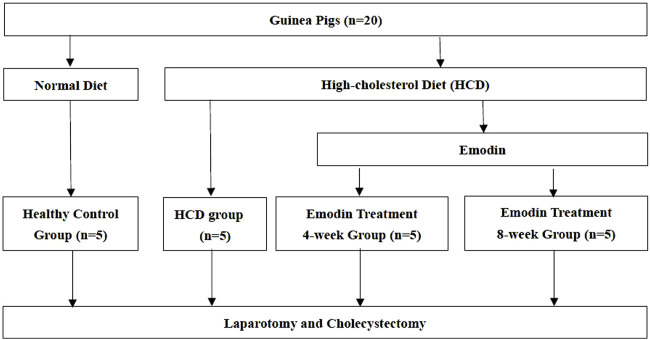
Study Flow Chart: Guinea pigs were randomly divided into four groups: one healthy control group and three study groups. The animals in the healthy control group were fed a normal diet. Animals in the study groups were fed a high-cholesterol diet (HCD) containing 2% cholesterol. After 8 weeks of feeding, animals in the study group were randomly divided into three groups: an HCD group, an emodin treatment 4-week group, and an emodin treatment 8-week group. Animals in the HCD group were fed a normal diet, while those in the emodin treatment groups were intraperitoneally injected with 2.5 mg/kg of emodin every other day for either 4 weeks (emodin treatment 4-week group) or 8 weeks (emodin treatment 8-week group).

Twenty clean guinea pigs, each weighing 100–120 g, were maintained under standard laboratory conditions at 23–26°C and 50%–70% humidity (Sinopharm Wuhan Institute of Biological Products Co., Ltd., Wuhan, China). After 7 days of adaptive feeding, the animals were randomly divided into four groups (n = 5): one healthy control group and three study groups. The animals in the healthy control group were fed a normal diet, whereas those in the study groups were fed a high-cholesterol diet (HCD) containing 2% cholesterol. All guinea pigs had free access to food and water. After 8 weeks of feeding, animals in the study group were randomly divided into three groups: an HCD group, an emodin treatment 4-week group, and an emodin treatment 8-week group. Animals in the HCD group were fed a normal diet, while those in the emodin treatment groups were intraperitoneally injected with 2.5 mg/kg of emodin (Sigma-Aldrich, St. Louis, United States) every other day for either another 4 weeks (emodin treatment 4-week group) or 8 weeks (emodin treatment 8-week group) (Figure 1). Animal experiments were approved by the Guangxi University of Chinese Medicine Institutional Welfare and Ethics Committee (No. DW 20230411-057).

### 2.2 Gallbladder tissue and serum pre-preparation

All animals underwent laparotomy and cholecystectomy under anesthesia with an intraperitoneal injection of 3% pentobarbital sodium (Shanghai Pharma New Asia Pharma, Shanghai, China). The gallbladder was incised along the neck to the fundus and washed three times with 1× phosphate-buffered saline (PBS) solution at 4°C to remove bile. Blood was aspirated from the guinea pig heart and centrifuged at 1,500 rpm for 5 min to separate serum.

### 2.3 Serum lipid test

Serum total cholesterol (TC) and triglyceride (TG) concentrations from three randomly selected animals from both healthy control and study groups were assessed using the ADVIA 400 Clinical Chemistry System (Siemens, Berlin, Germany).

### 2.4 Immunohistochemistry observation

After preparation, the gallbladder sample from one randomly selected animal in each group was fixed in 4% paraformaldehyde solution for 24 h. The samples were then embedded in paraffin. Approximately 5-μm-thick sections were cut and mounted on slides. Sections were treated with CD117/c-kit antibody (eBioscience, San Diego, CA, United States) at a 1:50 dilution and then incubated at 4°C for 12 h. Subsequently, the sections were incubated with appropriate secondary antibodies conjugated with horseradish peroxidase (HRP). Sections were then stained with diaminobenzidine and counterstained with hematoxylin. The stained sections were observed under the microscope (Olympus BX53; Tokyo, Japan), and three fields at ×400 magnification were selected randomly per section for ICC density detection.

### 2.5 Immunohistofluorescence and TUNEL assays

The gallbladder sample from one randomly selected animal in each group was prepared, embedded in paraffin, and sectioned. Fixed sections were dipped into 5% bovine albumin solution, followed by blocking for 30 min at 26°C. Subsequently, fixed sections were incubated overnight with a CD117/c-kit antibody (eBioscience, San Diego, CA, United States) at 4°C for 12 h. Sections were then incubated with the appropriate CY3-conjugated secondary antibody in 1× PBS at 37°C for 1 h.

TUNEL assays were used to detect gallbladder ICC apoptosis. The *In Situ* Cell Death Detection Kit, Fluorescein (Roche Applied Science, Mannheim, Germany), was used according to the manufacturer’s instructions. Stained sections were observed under the microscope (Olympus BX53; Tokyo, Japan), and three fields at ×400 magnification were selected randomly per section for apoptotic ICC density detection.

### 2.6 Transmission electron microscope observation

Gallbladder tissues from the healthy control, HCD, and emodin treatment 8-week groups were fixed and embedded in 2.5% glutaraldehyde solution at 4°C for 12 h after pre-preparation. Subsequently, samples were immersed in 1% osmium acid solution at 4°C for 2 h, followed by immersion in 2% uranyl acetate solution at 37°C for 1 h. Tissue dehydration was achieved using an ethanol solution, followed by a triple wash with an acetone solution. Subsequently, gallbladder samples were immersed in Epone 812 solution at 37°C for 12 h and dried at 60°C for 48 h. Subsequently, gallbladder samples were cut into 50 nm-thick sections and stained with uranyl acetate and lead citrate. The stained sections were observed using the transmission electron microscope (Hitachi H-600; Tokyo, Japan).

### 2.7 Western blotting analysis

Three gallbladder samples from each group were used for western blotting analysis. Proteins were extracted from 150 mg of gallbladder tissue from all samples using RIPA lysis buffer (Beyotime, Shanghai, China). Proteins were then separated by electrophoresis on 10% SDS sulfate-polyacrylamide gels and electrophoretically transferred onto a nitrocellulose membrane (Pierce Biotechnology, Inc., Rockford, IL, United States). Subsequently, all membranes were incubated with 5% BSA blocking buffer (Beyotime, Shanghai, China) at 26°C for 2 h and then incubated overnight at 4°C within anti-CD117/c-kit antibodies and anti-SCF antibodies (Abcam, Cambridge, United Kingdom) separately. Finally, the membranes were incubated with the appropriate HRP-conjugated secondary antibodies (Boster, Wuhan, China) at 37°C for 2 h. All protein bands were visualized using a chemiluminescence detection kit (ECL; Amersham, Pittsburgh, PA, United States) and X-ray film (Kadok China Investment Co. Ltd., Xiamen, China). Optical density analysis was performed using Band Scan 5.0 software (Glyko, Novato, California, United States).

### 2.8 Real-time polymerase chain reaction analysis

Three gallbladder samples from each group were used for real-time polymerase chain reaction analysis. Gallbladder tissues (100 mg) were obtained from all groups, and TRIzol reagent (Invitrogen, Carlsbad, California, United States) was used for extracting RNA. Subsequently, 3.674 µg of total RNA was reverse transcribed into cDNA and used as a template in the polymerase chain reaction. The semi-quantitative real-time polymerase chain reaction (RT-PCR) conditions were as follows: denaturation at 94°C for 4 min, followed by 30 cycles of annealing at 94°C for 30 s and extension at 56°C for 30 s; and incubation at 72°C for 4 min and 4°C for 4 min). The cDNA was diluted five times to conduct RT-PCR, with conditions as follows: 50°C for 2 min, initial denaturation at 95°C for 10 min, followed by 40 cycles at 95°C for 30 s and 60°C for 30 s.

The primers for GAPDH were 5′-ATC​ACT​GCC​ACC​CAG​AAG​ACT-3′ (forward) and 5′-CAG​ATC​CAC​AAC​CGA​CAC​ATT​A-3′ (reverse); the primers for SCF were 5′-GAA​AGA​TTC​CAG​AGT​CAG​TGT​CA-3′ (forward) and 5′-AAG​CAA​AGC​CAA​TCA​CAA​GAG-3′ (reverse); and the primers for c-kit were 5′-TAT​CCT​CCT​TAC​TCA​TGG​TCG​AA-3′ (forward) and 5′-TGC​AAT​GGA​ACT​TTC​AGC​CT-3′ (reverse).

### 2.9 Statistical analysis

SPSS for Windows version 25.0 (SPSS, Chicago, Illinois, United States) was used for the statistical analyses. Continuous variables were expressed as means ± standard deviation (*x* ± *s*). Categorical variables are presented as proportions and percentages. Comparisons of continuous variables were analyzed by first satisfying normality and homogeneity of variance, followed by analysis using the t-test. Categorical variables were compared using the chi-squared test or Fisher’s exact test. The results were considered statistically significant at a two-sided *P* < 0.05.

## 3 Results

### 3.1 Animal models and serum TC and TG testing

No animals died among the healthy control, HCD, or emodin-treated groups. As TC and TG were all reliable indicators of hypercholesterolemia, the serum lipid TC and TG levels were measured in the animal models. In the HCD group, TC and TG levels were significantly higher than those in the healthy control group (all *P* < 0.05), confirming the role of HCD in inducing cholelithiasis (Table 1).

**TABLE 1:** Serum lipid test during the high-cholesterol diet (mmol/L).

	Healthy control group	HCD group	
TC	0.90 ± 0.26	1.91 ± 0.24	t = −4.51, *p* = 0.04
TG	0.79 ± 0.07	1.93 ± 0.08	t = −49.36, *p* = 0.00

### 3.2 Immunohistochemistry observations

**FIGURE 2:**
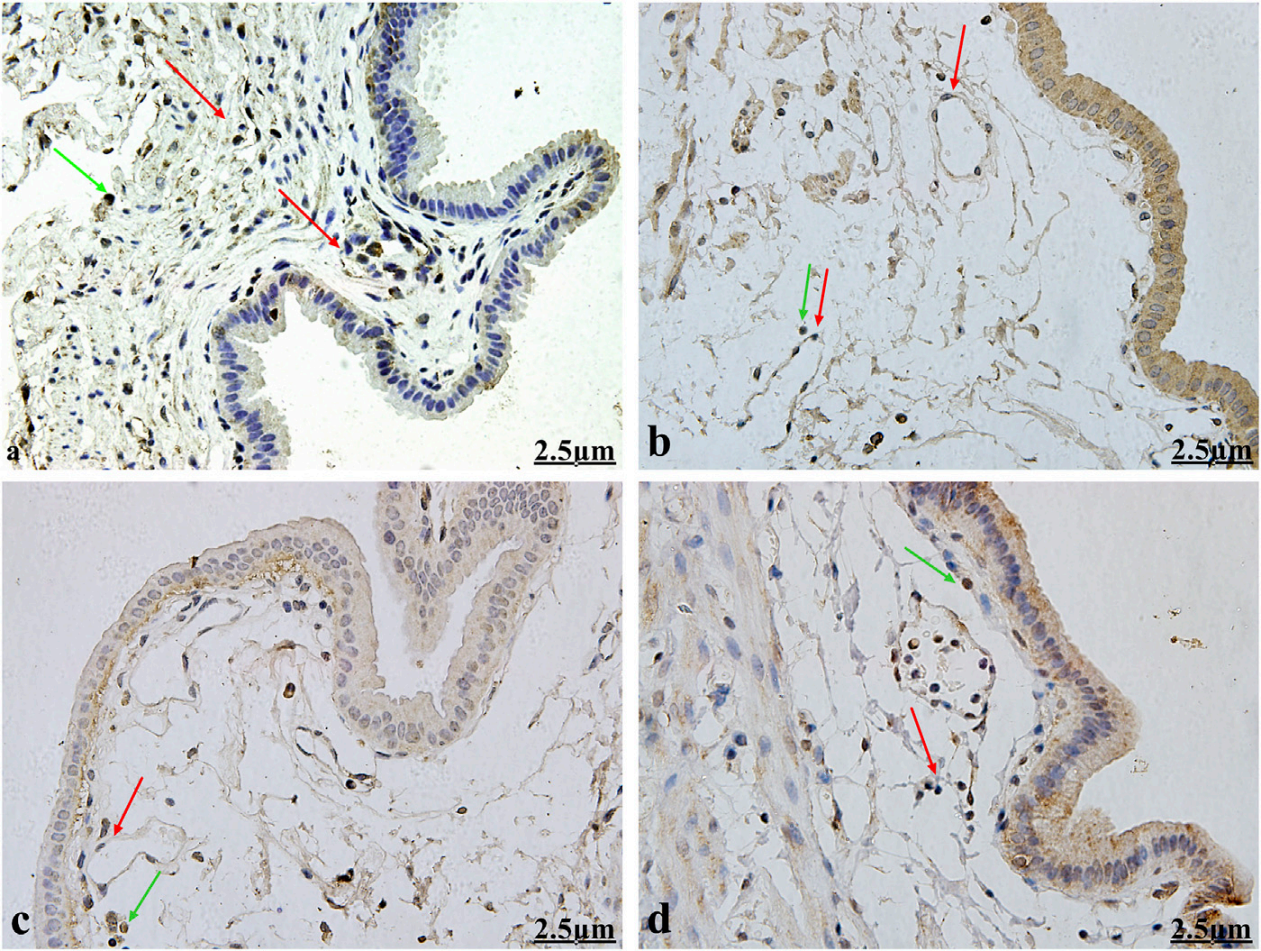
Immunohistochemical observation for gallbladder ICCs (×400). Gallbladder ICC surfaces (red arrows) displayed positive CD117/c-kit expression, appearing large with 2–5 slender and elongated processes. ICCs exhibited astrocyte-like or fusiform shapes, characterized by round or oval nuclei and minimal cytoplasm. They primarily inhabit the gallbladder muscular layer and are aligned parallel to gallbladder smooth muscle cells. They are typically found in small clusters of 2–3 cells interconnected by synapses and forming a net-like structure. Mast cells (green arrow) are single and round and distributed dispersedly. Figures 2A–D show the healthy control group, the HCD group, the emodin 4-week treatment group, and the emodin 8- week treatment group, respectively.

CD117/c-kit is a highly specific protein expressed on the surface of ICCs. Anti-CD117/c-kit antibody was used to detect gallbladder ICCs via immunohistochemistry. Following staining, the ICC surfaces were CD117/c-kit-positive, appearing large with 2–5 slender and elongated processes. These cells exhibited astrocyte-like or fusiform shapes, characterized by round or oval nuclei and minimal cytoplasm. Gallbladder ICCs primarily inhabit the gallbladder’s muscular layer, aligned parallel to gallbladder smooth muscle cells. Typically found in small clusters of 2–3 cells interconnected by synapses, gallbladder ICCs form a net-like structure. Occasional mast cells were also observed, which, like ICCs, displayed positive immunolabeling for CD117/c-kit. However, mast cells were single, round in shape, and distributed dispersedly (Figure 2).

### 3.3 Change in gallbladder ICC density

**FIGURE 3:**
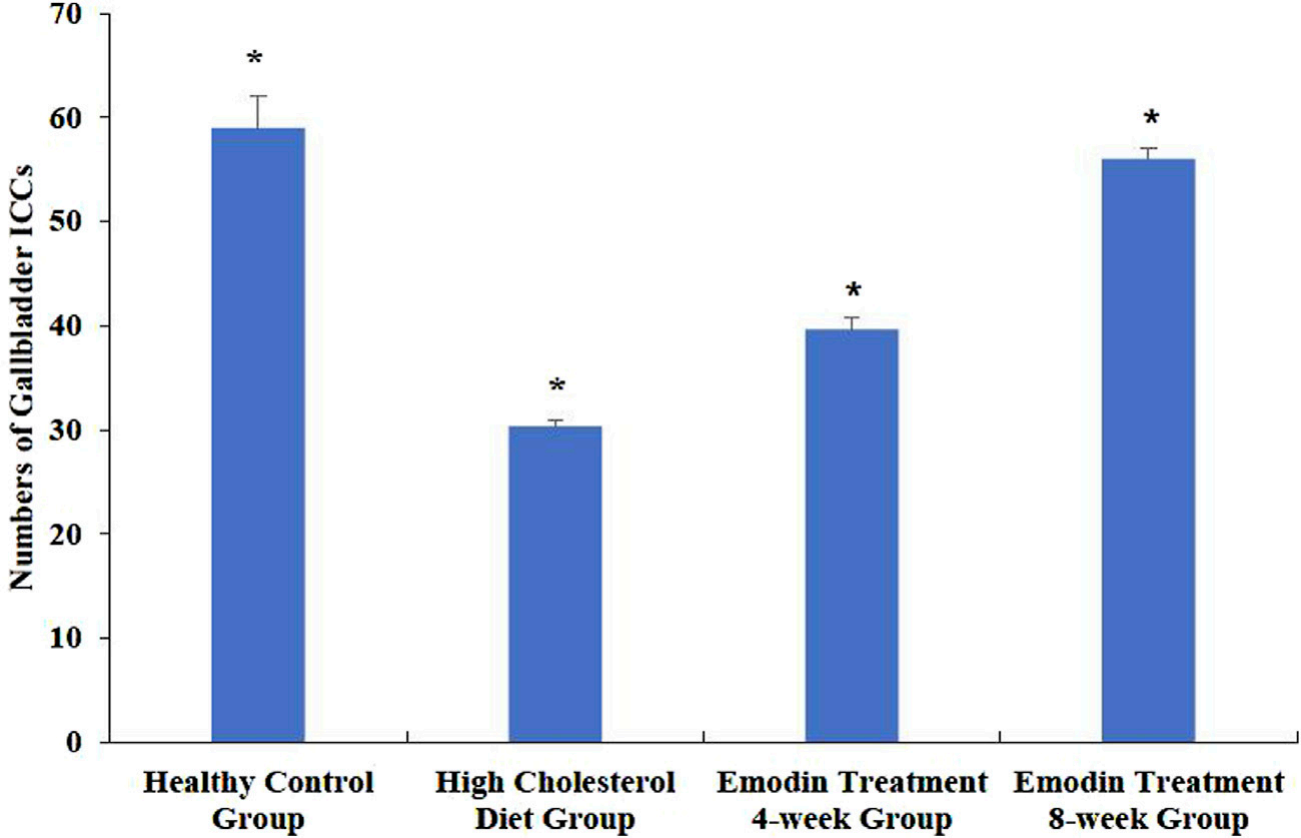
Changes in the numbers of gallbladder ICCs in the cholelithiasis and emodin treatment groups. Three ×400 views were selected randomly per section for ICC density detection. The density of gallbladder ICCs was assessed in the healthy control group, the HCD group, the emodin 4-week treatment group, and the emodin 8-week treatment group, yielding values of 59.00 ± 3.00, 30.33 ± 0.58, 39.67 ± 1.15, and 56.00 ± 1.00, respectively (*F = 190.048, *P* < 0.05).

The density of gallbladder ICCs decreased in cases of cholelithiasis but gradually recovered following emodin treatment. Random fields were selected using light microscopy to quantify gallbladder ICC density. The density of gallbladder ICCs was assessed in the healthy control, HCD, emodin treatment 4-week, and emodin treatment 8-week groups, yielding values of 59.00 ± 3.00, 30.33 ± 0.58, 39.67 ± 1.15, and 56.00 ± 1.00, respectively (*F* = 190.048, *P* < 0.05) (Figure 3).

### 3.4 Changes in gallbladder ICC apoptosis

**FIGURE 4:**
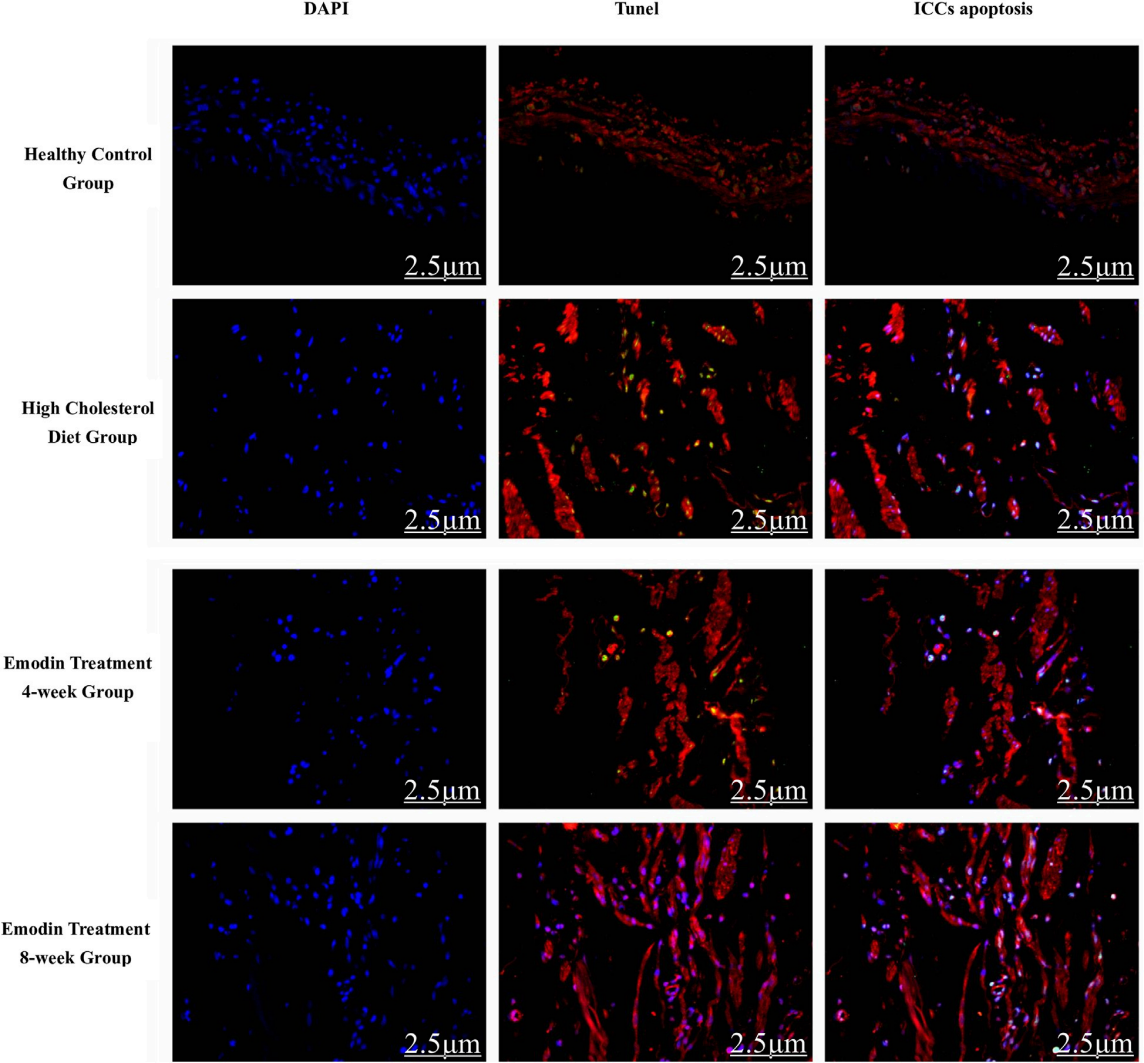
Gallbladder ICC apoptosis in cholelithiasis and emodin treatment (×400). The quantity of apoptotic gallbladder ICCs escalated during cholelithiasis, gradually diminishing following emodin treatment.

**FIGURE 5:**
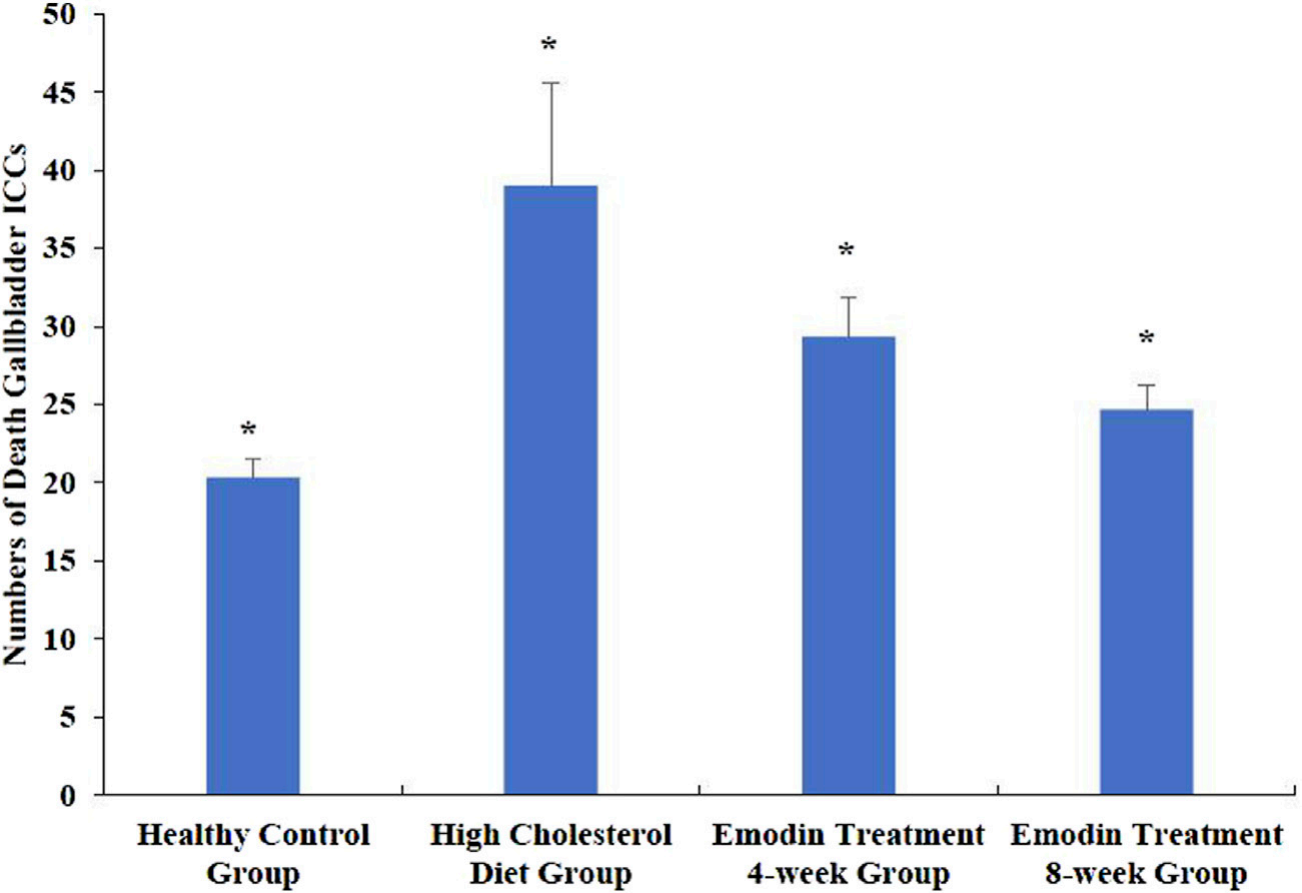
Changes in the numbers of gallbladder ICC deaths in cholelithiasis and emodin treatment. Three ×400 views were randomly selected per section for apoptotic ICC density detection. The densities of apoptotic ICCs in the healthy control group, the HCD group, the emodin 4-week treatment group, and the emodin 8-week treatment group were 20.33 ± 1.15, 39.00 ± 6.56, 29.33 ± 2.52, and 24.67 ± 1.53, respectively (*F = 14.507, *P* < 0.05)

The number of apoptotic gallbladder ICCs increased during cholelithiasis and gradually diminished following emodin treatment (Figure 4). The densities of apoptotic ICCs in the healthy control, HCD, emodin treatment 4-week, and emodin treatment 8-week groups were 20.33 ± 1.15, 39.00 ± 6.56, 29.33 ± 2.52, and 24.67 ± 1.53, respectively (*F* = 14.507, *P* < 0.05) (Figure 5).

### 3.5 Morphological characteristics of gallbladder ICCs

TEM analysis unveiled the distribution of gallbladder ICCs in the subepithelial connective tissue layer and muscularis propria in the healthy control group. These cells exhibited fusiform or astrocytic shapes, with abundant cytoplasm and large, round nuclei, consistent with observations of CD117/c-kit immunohistochemistry and immunofluorescence.

**FIGURE 6:**
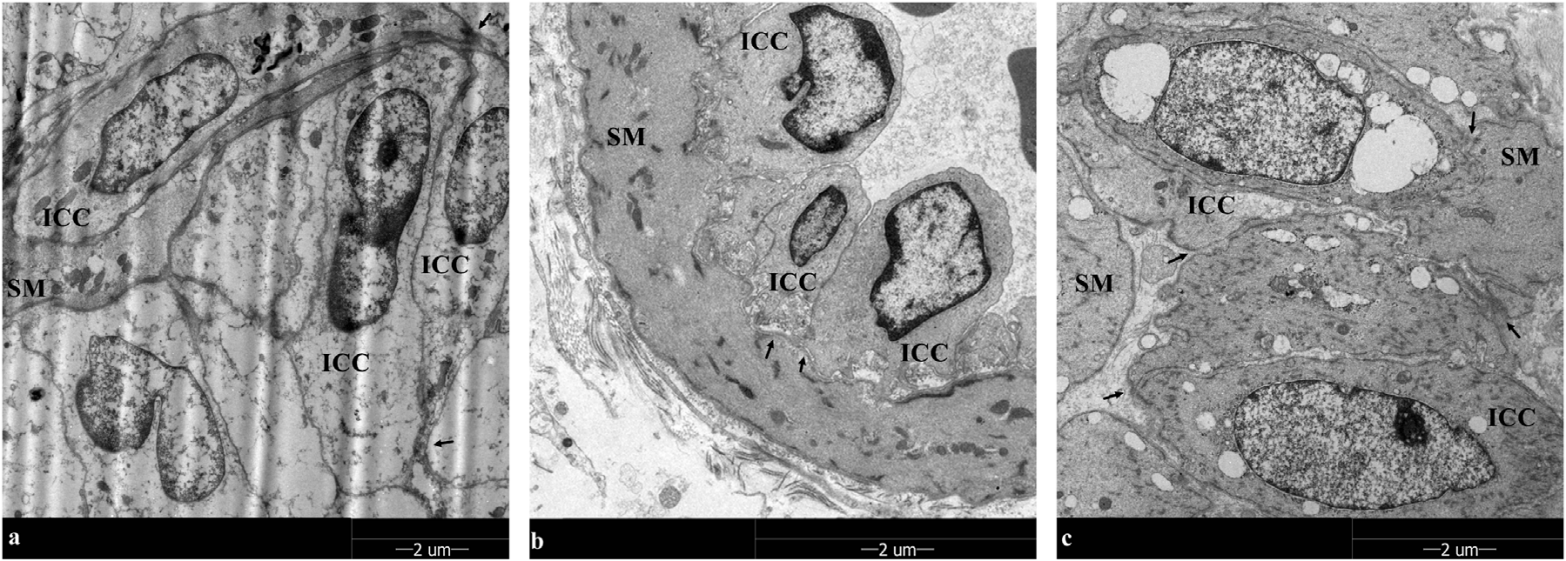
TEM observation in gallbladder ICCs (×1700): Gallbladder ICC cells exhibited fusiform or astrocytic shapes, with abundant cytoplasm and large, round nuclei in smooth muscle (SM), and the fewer gallbladder ICCs were interconnected by synapses. In addition, the net-like structure in gallbladder ICCs disappeared during cholelithiasis. After 8 weeks of emodin treatment, the cell-to-cell synapse connections and net-like structure of the gallbladder ICCs had recovered. **(A–C)** are the healthy control group, the HCD group, and the emodin 8-week treatment group, respectively. Gallbladder ICC cell-to-cell synapse connections interconnections are marked by arrows.

However, under TEM, a reduction in the number of gallbladder ICCs interconnected by synapses was noted, along with the dissolution of the net-like structure in gallbladder ICCs during cholelithiasis. After 8 weeks of emodin treatment, the cell-to-cell synaptic connections and net-like structure of the gallbladder ICCs had recovered (Figure 6).

### 3.6 Expression level changes of gallbladder tissue SCF and c-kit proteins

During cholelithiasis, the expression levels of gallbladder tissue SCF and c-kit proteins decreased, which increased gradually following emodin treatment. Notably, after 8 weeks of treatment, gallbladder SCF and c-kit protein expression exhibited a significant increase compared to that after 4 weeks of treatment.

**FIGURE 7:**
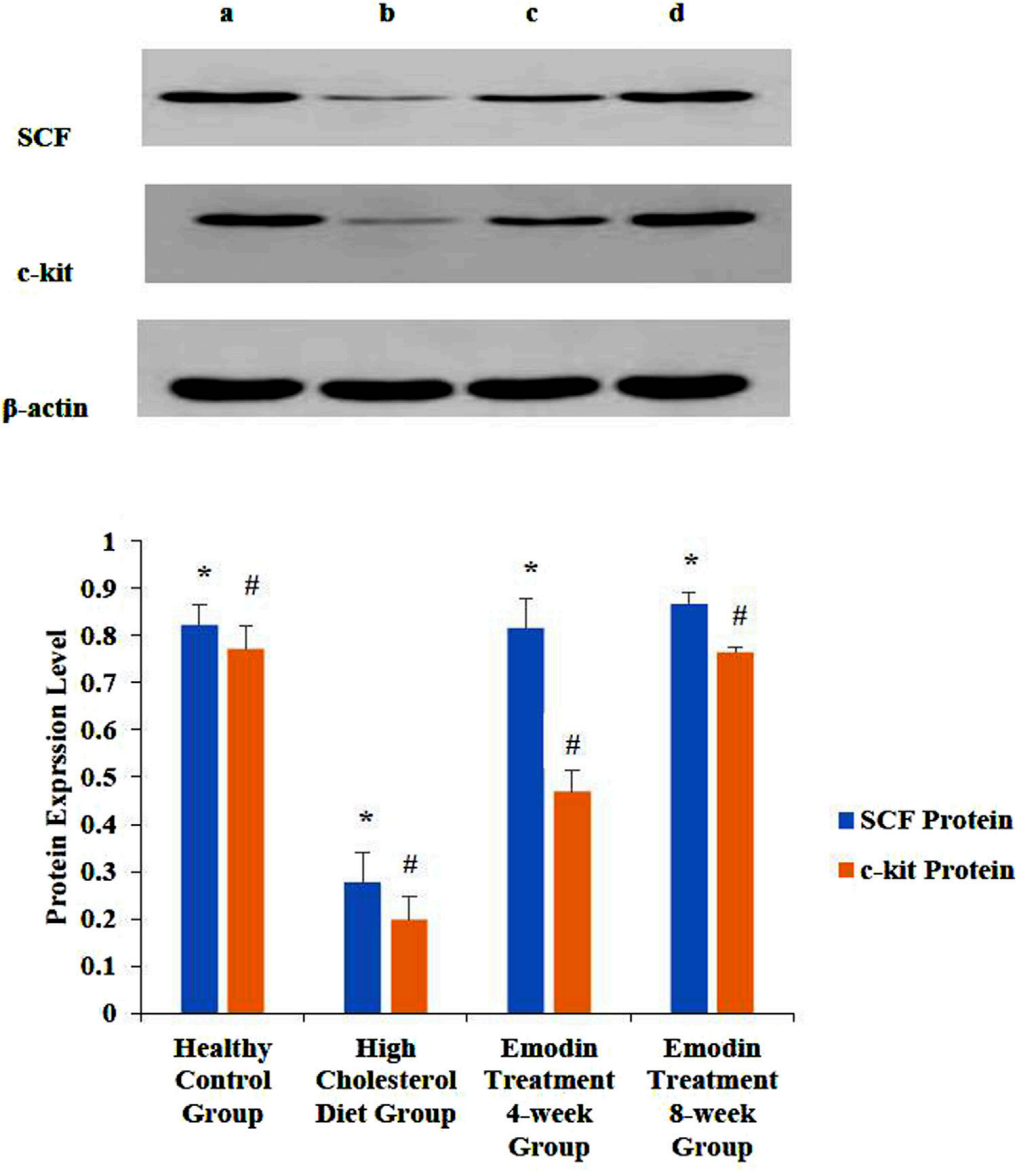
Expression level change of gallbladder SCF and c-kit protein: Three gallbladder samples from each group were analyzed using protein detection. The ratios of SCF to β-actin in the healthy control group, the HCD group, the emodin 4-week treatment group, and the emodin 8-week treatment group were 0.82 ± 0.04, 0.28 ± 0.06, 0.82 ± 0.06, and 0.87 ± 0.02, respectively (*F = 77.513, *P* < 0.05). Similarly, the ratios of c-kit to β-actin in the healthy control groups, the HCD group, the emodin 4-week treatment group, and the emodin 8-week treatment group were 0.77 ± 0.05, 0.20 ± 0.05, 0.47 ± 0.05, and 0.76 ± 0.01, respectively (#F = 129.811, *P* < 0.05). Figures 7A–D show the healthy control group, the HCD group, the emodin 4-week treatment group, and the emodin 8- week treatment group, respectively.

The ratios of SCF to β-actin in the healthy control, HCD, emodin treatment 4-week, and emodin treatment 8-week groups were 0.82 ± 0.04, 0.28 ± 0.06, 0.82 ± 0.06, and 0.87 ± 0.02, respectively (*F* = 77.513, *P* < 0.05). Similarly, the ratios of c-kit to β-actin in the healthy control, HCD, emodin treatment 4-week, and emodin treatment 8-week groups were 0.77 ± 0.05, 0.20 ± 0.05, 0.47 ± 0.05, and 0.76 ± 0.01, respectively (*F* = 129.811, *p* < 0.05) (Figure 7).

### 3.7 Expression levels of gallbladder tissue SCF mRNA and c-kit mRNA

The expression levels of gallbladder tissue SCF mRNA and c-kit mRNA decreased in cholelithiasis, while the levels of SCF mRNA and c-kit mRNA gradually increased after emodin treatment.

**FIGURE 8:**
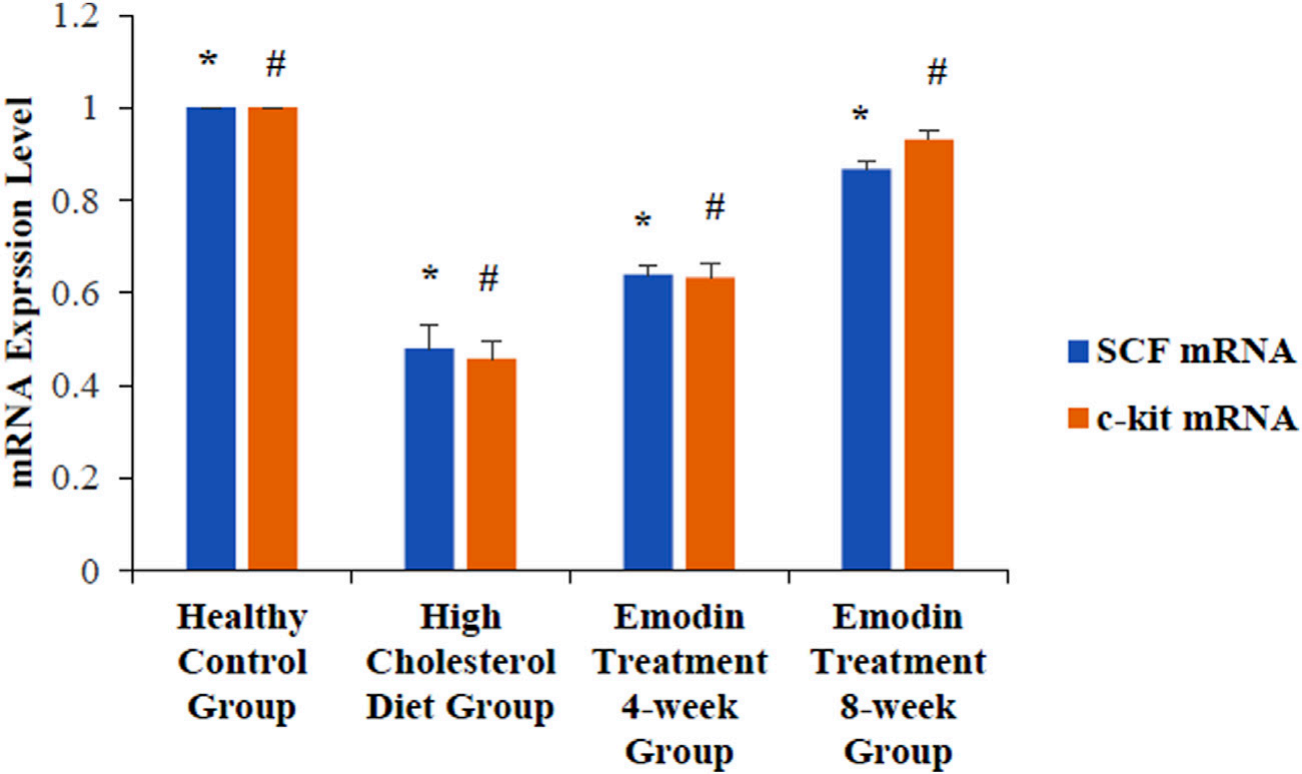
Expression level change of gallbladder SCF and c-kit mRNA: Three gallbladder samples from each group were analyzed using mRNA detection. The levels of gallbladder tissue SCF mRNA in the healthy control group, the HCD group, the emodin 4-week treatment group, and the emodin 8-week treatment group were 1.00 ± 0.00, 0.48 ± 0.05, 0.64 ± 0.02, and 0.87 ± 0.02, respectively (*F = 62.125, *P* < 0.05). The levels of c-kit mRNA in the healthy control group, the HCD group, the emodin 4-week treatment group, and the emodin 8-week treatment group were 1.00 ± 0.00, 0.46 ± 0.04 0.64 ± 0.02, and 0.93 ± 0.02, respectively (#*F* = 101.87, *P* < 0.05).

The levels of gallbladder tissue SCF mRNA in the healthy control, HCD, emodin 4-week treatment, and emodin treatment 8-week groups were 1.00 ± 0.00, 0.48 ± 0.05, 0.64 ± 0.02, and 0.87 ± 0.02, respectively (*F* = 62.125, *P* < 0.05). The levels of c-kit mRNA in the healthy control, HCD, emodin treatment 4-week, and emodin treatment 8-week groups were 1.00 ± 0.00, 0.46 ± 0.04 0.64 ± 0.02, and 0.93 ± 0.02, respectively (*F* = 101.87, *P* < 0.05) (Figure 8).

## 4 Discussion

The progression of cholelithiasis is influenced by various risk factors, including biliary cholesterol hypersaturation and motility disorders. Moreover, hypercholesterolemia, including increased serum levels of TC and TG, induces cholelithiasis (Di Ciaula A et al., 2019; Huang ZP et al., 2018), which is consistent with that obtained in the present study. A previous study established the involvement of gallbladder ICCs in the development of cholelithiasis (Fu BB et al., 2021). Moreover, our previous investigations confirmed a decrease in gallbladder ICC number and an increase in apoptotic gallbladder ICCs during cholelithiasis (Huang ZP et al., 2018). Additionally, the downregulation of the SCF/c-kit pathway has been observed in cholelithiasis (Fan Y et al., 2014).

Several studies have suggested possible mechanisms underlying cholelithiasis and motility disorders. First, oxidative stress, changes in bile composition, and inflammatory responses downregulate the SCF/c-kit pathway, resulting in the loss of gallbladder ICCs during cholelithiasis, which decreases gallbladder motility (Grover M et al., 2019; Pasternak A et al., 2017; Wan JF et al., 2018). Second, the lower response of gallbladder ICCs to CCK can also decrease gallbladder motility in cholelithiasis (Fan Y et al., 2015; Xu D et al., 2008).

Recent studies have suggested that emodin has diverse pharmacological activities, including anti-inflammatory, antioxidant, neuroprotective, and immunosuppressive properties (Liu Y et al., 2022; Liu YP et al., 2023; Liu YX et al., 2009; Mitra S et al., 2022). Its pharmacological effect would be through the TGF-β1/Smad and NF-κB signaling pathways, similar to another natural herbal medicine, *Curcumae rhizoma* (Gao TH et al., 2022; Liu B et al., 2020; Ma L et al., 2018). It has been identified as a modulator of gallbladder muscle contractility, enhancing its contractile function (Wu ZX et al., 2009). Recent studies have confirmed that emodin enhances the contractility of the gallbladder muscle by regulating CCK, Ca^2+^, and the Gs, Gi, and Cap signaling pathways (Fang BJ et al., 2016). In addition, emodin inhibits voltage-dependent K^+^ currents via the protein kinase C pathway and increases Ca^2+^ influx through L-type calcium channels via the PKC pathway (Wu ZX et al., 2008; Wu ZX et al., 2009). Our study also indicates that emodin regulates the SCF/c-kit pathway, both at the protein and mRNA levels, and alters the number of gallbladder ICCs to enhance gallbladder muscle contractility.

Recent findings confirm that oxidative stress and inflammatory responses contribute to the downregulation of the SCF/c-kit pathway, causing injury to the network structure of gallbladder ICCs, reducing their numbers, and ultimately leading to gallbladder dysfunction in cholelithiasis (Liu W et al., 2023; López-Pingarrón L et al., 2023; Xie Y et al., 2021). Our findings suggest that emodin would upregulate the SCF/c-kit pathway, restore the network structure of gallbladder ICCs, increase the number of ICCs, and reduce gallbladder dysfunction through its anti-inflammatory and anti-oxidative stress properties.

There were several limitations in this study. First, only animal experiments have been conducted in this study; cell culture experiments and clinical studies should be conducted to explore the effect of emodin on gallbladder motility. Second, the mechanism underlying the effect of emodin on gallbladder motility is still unclear; further investigation is warranted to elucidate the additional mechanisms involved.

In conclusion, this study shows that cholelithiasis downregulates the SCF/c-kit pathway, injuring gallbladder ICCs. Emodin upregulates the SCF/c-kit pathway, restores the number and network structure of gallbladder ICCs, and might contribute to recovery from gallbladder motility disorders.

## Data Availability

The original contributions presented in the study are included in the article/Supplementary Material; further inquiries can be directed to the corresponding author.
